# A New Model for Complex Dynamical Networks Considering Random Data Loss

**DOI:** 10.3390/e21080797

**Published:** 2019-08-15

**Authors:** Xu Wu, Guo-Ping Jiang, Xinwei Wang

**Affiliations:** 1 School of Automation, Nanjing University of Posts and Telecommunications, Nanjing 210023, China; 2 Jiangsu Engineering Lab for IOT Intelligent Robots(IOTRobot), Nanjing 210023, China

**Keywords:** complex dynamical network, random data loss, Lyapunov stability theory, stochastic analysis method

## Abstract

Model construction is a very fundamental and important issue in the field of complex dynamical networks. With the state-coupling complex dynamical network model proposed, many kinds of complex dynamical network models were introduced by considering various practical situations. In this paper, aiming at the data loss which may take place in the communication between any pair of directly connected nodes in a complex dynamical network, we propose a new discrete-time complex dynamical network model by constructing an auxiliary observer and choosing the observer states to compensate for the lost states in the coupling term. By employing Lyapunov stability theory and stochastic analysis, a sufficient condition is derived to guarantee the compensation values finally equal to the lost values, namely, the influence of data loss is finally eliminated in the proposed model. Moreover, we generalize the modeling method to output-coupling complex dynamical networks. Finally, two numerical examples are provided to demonstrate the effectiveness of the proposed model.

## 1. Introduction

Complex networks exist in different fields such as Internet, power grids, food web, etc., and have received a great deal attention over the past decades. Researchers have tried to build mathematical models for various types of networks in the real world, some of which focused on the network topology. These complex network models have been studied by graph theory, which are represented by nodes connected by edges. Some classic models, such as the E-R random-graph model [1], the WS small-world model [2], and the BA scale-free model [3], have led research hotspots in complex networks and made outstanding contributions to the development of complex networks.

Subsequently, some researchers realized that they should not only focus on the network’s topological connectivity, but should also consider the dynamics of network nodes in order to better understand the dynamical behaviors of various complex networks. Pecora and Carroll [4] constructed a state-coupling model by introducing coupling coefficients and a matrix to link the node connectivity and the node dynamics together. Wang et al. [5] considered a scale-free dynamical network consisting of identical linearly coupled nodes and studied its robustness and fragility of synchronization. Lü et al. [6] introduced a time-varying state-coupling complex dynamical network model, with time-varying coupling configuration matrix and inner-coupling matrix. Differing from the previous studies, Li et al. [7] restricted the inner-coupling matrix as the identity matrix, which means that two coupled nodes are diagonally linked through their corresponding components. These state-coupling complex dynamical network models have been adopted by many follow-up studies.

Many practical complex networks, covering different fields such as communication networks and social networks [8,9], all undertake the objective of information transmission. Especially for communication networks, information is the most fundamental element. Some application areas (e.g., cell phones and aerospace) make great demands on the reliability of the data transmission. Therefore, a number of studies have considered the actual situation involving unreliable factors such as noise [10,11,12], time delay [13,14,15,16], and data loss [17,18,19,20,21,22], and investigated the influence of these unreliable factors on the complex networks.

Due to network congestion or node failures, data loss is a common phenomenon in data transmission in complex dynamical networks. Yang et al. [23] developed a model for complex dynamical networks with random packet losses which occur in the communication links between every two neighbor nodes. In their paper, the packet losses are described by a set of Bernoulli random variables multiplied by coupling coefficients, and the exponential mean-square stability and synchronization problems are investigated by defining the packet loss probability matrix (PLPM) [23]. However, there is no treatment of the random packet loss in the interaction topology. Even using for reference the previous data compensation methods that are applied in different research areas such as filtering and stability in networked systems [24,25,26,27], estimation in complex dynamical networks [28,29], etc., there are still some differences between the compensation values and the actual values (except for stationary systems)—that is, the influence of data loss still exists in the network.

Based on the above, it is clear that complex dynamical network models considering random data loss in the interaction topology require further investigation. Therefore, in this paper, we consider a discrete-time state-coupling complex dynamical network with random data loss on the interactions between the neighbor nodes, and a new complex dynamical network model is presented by introducing an auxiliary observer. When the data loss takes place in a communication channel from one node to another at one moment, the corresponding data in the observer will be used to compensate for the lost data in the coupling term. Applying Lyapunov stability theory and stochastic analysis, we derive a sufficient condition in the form of LMIs to guarantee that the compensation values finally equal to the lost values—namely, the influence of random data loss will be eliminated in the proposed model.The output-coupling complex dynamical network [30] is another model which is recognized and studied by researchers. Here, we generalize the proposed modeling method to output-coupling complex dynamical networks.

The remainder of this paper is organized as follows. A model for discrete-time complex dynamical networks with random data loss is formulated in Section 2. In Section 3, the model analysis is presented. Some numerical examples are provided to demonstrate the effectiveness of the proposed model in Section 4. Conclusions are given in Section 5.

**Notation** **1.** *Unless specified otherwise, throughout this paper we let $∥x∥$ denote the Euclidean norm of a vector x. I is an identity matrix of suitable dimensions and O, is a zero matrix of suitable dimensions. $X^{T}$ represents the transpose of a matrix X, and X>0, X<0, and X≤0 mean that X is positive-definite, negative-definite, and negative-semidefinite, respectively. ${\left[X\right]}_{N\times N}$ is a N×N block matrix whose every block is X. $E\left[\cdot \right]$ denotes the operator of the mathematical expectation. *⊗* denotes the Kronecker product, and *∘* denotes the Hadamard product. *∗* denotes the transpose of symmetric term and $diag\left(\cdots \right)$ denotes a block-diagonal matrix.*

## 2. Network Modeling and Preliminaries

The typical discrete-time complex dynamical network is as follows:

(1) $$\begin{array}{c} x_{i,k+1}=Ax_{i,k}+f\left(x_{i,k}\right)+d\sum_{j=1}^{N}c_{ij}\Gamma x_{j,k}, \end{array}$$

where i=1,2,…,N denotes the *i*th node, $x_{i,k}={\left(x_{i1,k},x_{i2,k},\ldots ,x_{in,k}\right)}^{T}\in R^{n}$ denotes the state vector of the *i*th node at time *k*, $A\in R^{n\times n}$ is a constant matrix, $f\left(\cdot \right):R^{n}\to R^{n}$ is the known nonlinear function, *d* is the coupling strength, and matrix $C={\left(c_{ij}\right)}_{N\times N}$ is the coupling configuration matrix. If there is a link from node *j* to node $i\left(i\neq j\right)$, then $c_{ij}=1$; otherwise, $c_{ij}=0$. Assume that matrix *C* satisfies $c_{ii}=-\sum_{j=1,j\neq i}^{N}c_{ij}$. $\Gamma \in R^{n\times n}$ is the inner connecting matrix between two connected nodes.

The complex dynamical network model (1) is established in an ideal situation without the consideration of random data loss. Yang et al. [23] considered the data loss taking place in the communication between neighbor nodes, and established the corresponding complex dynamical network model as follows:

(2) $$\begin{array}{c} x_{i,k+1}=Ax_{i,k}+f\left(x_{i,k}\right)+d\sum_{j=1}^{N}b_{ij,k}c_{ij}\Gamma x_{j,k}, \end{array}$$

where $b_{ij,k}\in R$ are independent identically distributed Bernoulli random variables. If there is data loss in the link from node *j* to node $i\left(i\neq j\right)$ at time *k*, then $b_{ij,k}=0$; otherwise, $b_{ij,k}=1$. $b_{ii,k}=1$ always holds. $b_{ij,k}$ takes 0 or 1 with the probabilities:

$$\begin{array}{c} Pr\left\{b_{ij,k}=1\right\}=E\left\{b_{ij,k}\right\}={\bar{b}}_{ij},\\ Pr\left\{b_{ij,k}=0\right\}=1-{\bar{b}}_{ij}={\hat{b}}_{ij}. \end{array}$$

$\bar{B}={\left({\bar{b}}_{ij}\right)}_{N\times N}$ and $\hat{B}={\left({\hat{b}}_{ij}\right)}_{N\times N}$.

However, the influence of data loss still exists in the network, and may give rise to adverse effects such as low transmission efficiency or transmission failure. Therefore, we model a complex dynamical network with data loss by introducing an auxiliary observer and compensating for the lost states with the corresponding observer states in the coupling term. The corresponding state-coupling complex dynamical network model is presented as follows:

(3) $$\begin{array}{c} x_{i,k+1}=Ax_{i,k}+f\left(x_{i,k}\right)+d\sum_{j=1}^{N}\left[b_{ij,k}c_{ij}\Gamma x_{j,k}+\left(1-b_{ij,k}\right)c_{ij}\Gamma {\hat{x}}_{j,k}\right],\\ y_{i,k}=H_{i}x_{i,k},\\ {\hat{x}}_{i,k+1}=A{\hat{x}}_{i,k}+f\left({\hat{x}}_{i,k}\right)+d\sum_{j=1}^{N}c_{ij}\Gamma {\hat{x}}_{j,k}+K_{i}\left({\hat{y}}_{i,k}-y_{i,k}\right),\\ {\hat{y}}_{i,k}=H_{i}{\hat{x}}_{i,k}, \end{array}$$

where $y_{i,k}\in R^{m}$ are the outputs of the *i*th node in the network, $H_{i}\in R^{m\times n}$ denote the output matrices of the *i*th node, ${\hat{x}}_{i,k}={\left({\hat{x}}_{i1,k},{\hat{x}}_{i2,k},\ldots ,{\hat{x}}_{in,k}\right)}^{T}\in R^{n}$ denote the observation values of $x_{i,k}$, ${\hat{y}}_{i,k}\in R^{m}$ are the outputs of the observer, and $K_{i}\in R^{n\times m}$ are the observer gains to be determined. Here, we assume the data loss processes on all the interactions between the neighbor nodes are mutually independent.

**Remark** **1.** *The random data losses bring uncertainty to the network, which can be measured by the entropy in information theory [31,32]. In this paper, the random data losses are described by a set of random variables satisfying the Bernoulli distribution, so the entropy of each Bernoulli random process is $H\left(b_{ij}\right)=-Pr\left\{b_{ij}=1\right\}\log Pr\left\{b_{ij}=1\right\}-Pr\left\{b_{ij}=0\right\}\log Pr\left\{b_{ij}=0\right\}=-{\bar{b}}_{ij}\log{\bar{b}}_{ij}-{\hat{b}}_{ij}\log{\hat{b}}_{ij}$. Since all the Bernoulli random processes are mutually independent, the joint entropy is $\sum_{i,j=1}^{N}c_{ij}H\left(b_{ij}\right)$.*

**Remark** **2.** *The model (3) is constructed under the assumption that the transmission time between the network and the observer can be neglected. Namely, the transmission time from $y_{i,k}$ to the observer and ${\hat{x}}_{j,k}$ to the network is neglectable.*

**Remark** **3.** *In order to eliminate the influence of random data loss, we tried to find a kind of compensation method whose compensation value could finally equal to the lost data. Because of the favorable performance on observation, we chose the observer state values as the compensation values and propose the complex dynamical network model (3). Note that the aim of the proposed model (3) is to compensate the lost network states with the observer states after the error convergence is achieved in an unbiased fashion in real-time. The data loss existing before convergence cannot be compensated without bias. We will study the finite-time and the fixed-time asymptotic convergence in future work to speed up convergence.*

**Remark** **4.** *In the actual data transmission process, there generally exists a detecting mechanism to judge whether the data are transmitted successfully or not. For example, in the Internet, TCP (Transmission Control Protocol) uses the ACK (acknowledgment character) to acknowledge receipt of a packet. Therefore, we can judge whether $b_{ij,k}=1$ or not via a certain detection mechanism and realize the construction of the proposed model.*

For the purpose of analyzing the proposed complex dynamical network model (3), an assumption and a lemma are given as follows.

**Assumption** **1.** *There exists a positive constant α such that*

(4) $$∥f\left(u\right)-f\left(v\right)∥\leq \alpha ∥u-v∥\;\;\;\;\forall u,v\in R^{n}.$$

**Lemma** **1** ([33])**.** *Given real matrices $\Omega_{1}$, $\Omega_{2}$, and $\Omega_{3}$ of appropriate dimensions, let $\Omega_{1}=\Omega_{1}^{T}$, and $\Omega_{2}=\Omega_{2}^{T}>0$. Then, the linear matrix inequality $\Omega_{1}+\Omega_{3}^{T}\Omega_{2}^{-1}\Omega_{3}<0$ holds if and only if the following condition holds:*

$$\left[\begin{array}{cc} \Omega_{1} & \Omega_{3}^{T}\\ \Omega_{3} & -\Omega_{2} \end{array}\right]<0.$$

## 3. Model Analysis

In this section, we analyze the complex dynamical network model (3) proposed in Section 2.

An auxiliary observer is introduced in the proposed model (3). Once the data loss happens in a data transmission channel, the corresponding data in the observer will be used to compensate for the lost data. If appropriate observer gains $K_{i}$ are chosen to make the observation states ${\hat{x}}_{i,k}$ approach the network states $x_{i,k}$, then the observer outputs ${\hat{y}}_{i,k}$ will approach the network outputs $y_{i,k}$, the coupling term $\sum_{j=1}^{N}\left[b_{ij,k}c_{ij}\Gamma x_{j,k}+\left(1-b_{ij,k}\right)c_{ij}\Gamma {\hat{x}}_{j,k}\right]$ will approach $\sum_{j=1}^{N}c_{ij}\Gamma x_{j,k}$, and the output feedback term $K_{i}\left({\hat{y}}_{i,k}-y_{i,k}\right)$ will approach the zero matrix. Thus the proposed complex dynamical network model (3) will approach the ideal complex dynamical network model (1), that is, the influence of the random data loss can be eliminated when state observation is achieved.

In the following, we derive a method to determine feasible observer gains $K_{i}$.

Defining $e_{i,k}={\hat{x}}_{i,k}-x_{i,k}\left(i=1,2,\ldots ,N\right)$ as the observation errors in the complex dynamical network model (3), one can obtain the following error system:

(5) $$\begin{array}{cc} e_{i,k+1} & =A{\hat{x}}_{i,k}-Ax_{i,k}+f\left({\hat{x}}_{i,k}\right)-f\left(x_{i,k}\right)+K_{i}\left({\hat{y}}_{i,k}-y_{i,k}\right)+d\sum_{j=1}^{N}c_{ij}\Gamma {\hat{x}}_{j,k}\\ & \;\;\;\;-d\sum_{j=1}^{N}\left[b_{ij,k}c_{ij}\Gamma x_{j,k}+\left(1-b_{ij,k}\right)c_{ij}\Gamma {\hat{x}}_{j,k}\right]\\ & =Ae_{i,k}+f\left({\hat{x}}_{i,k}\right)-f\left(x_{i,k}\right)+K_{i}H_{i}e_{i,k}+d\sum_{j=1}^{N}\left[b_{ij,k}c_{ij}\Gamma {\hat{x}}_{j,k}+\left(1-b_{ij,k}\right)c_{ij}\Gamma {\hat{x}}_{j,k}\right]\\ & \;\;\;\;-d\sum_{j=1}^{N}\left[b_{ij,k}c_{ij}\Gamma x_{j,k}+\left(1-b_{ij,k}\right)c_{ij}\Gamma {\hat{x}}_{j,k}\right]\\ & ={\tilde{f}}_{i,k}+\left(A+K_{i}H_{i}\right)e_{i,k}+d\sum_{j=1}^{N}b_{ij,k}c_{ij}\Gamma e_{j,k}, \end{array}$$

where ${\tilde{f}}_{i,k}=f\left({\hat{x}}_{i,k}\right)-f\left(x_{i,k}\right)$.

**Theorem** **1.** *Suppose that Assumption 1 holds. The error system (5) is asymptotically stable and the error states converge to zero if there exist matrices $P_{i}=P_{i}^{T}>0$, $S_{i}\left(i=1,2,\ldots ,N\right)$ and a scalar τ>0 satisfying the following inequality:*

(6) $$\left[\begin{array}{ccc} \Psi +\tau \alpha^{2}I_{N\cdot n} & M+dG^{T}\Lambda & Q\\ M^{T}+d\Lambda G & \Lambda -\tau I_{N\cdot n} & O\\ Q^{T} & O & -\Lambda \end{array}\right]<0,$$

*where*

*$\Psi ={\bar{A}}^{T}\Lambda \bar{A}+Q\bar{A}+{\bar{A}}^{T}Q^{T}+dMG+dG^{T}M^{T}+d^{2}\Phi -\Lambda$,*

*$S_{i}=P_{i}K_{i}$, $\bar{A}=I_{N}⊗A$, $\Lambda =diag\left(P_{1},\ldots ,P_{N}\right)$,*

*$Q=diag\left(H_{1}^{T}S_{1}^{T},\ldots ,H_{N}^{T}S_{N}^{T}\right)$, $M={\bar{A}}^{T}\Lambda +Q$,*

*$G=\left(\left(\bar{B}\circ C\right)⊗I_{n}\right){\left[\Gamma \right]}_{N\times N}$,*

*$\Phi =\left[\begin{array}{cccc} \sum_{i=1}^{N}{\bar{b}}_{i1}c_{i1}^{2}\Gamma^{T}\;P_{i}\Gamma & \sum_{i=1}^{N}{\bar{b}}_{i1}c_{i1}\Gamma^{T}P_{i}{\bar{b}}_{i2}c_{i2}\Gamma & \cdots & \sum_{i=1}^{N}{\bar{b}}_{i1}c_{i1}\Gamma^{T}P_{i}{\bar{b}}_{iN}c_{iN}\Gamma \\ {}* & \sum_{i=1}^{N}{\bar{b}}_{i2}c_{i2}^{2}\Gamma^{T}P_{i}\Gamma & \cdots & \sum_{i=1}^{N}{\bar{b}}_{i2}c_{i2}\Gamma^{T}P_{i}{\bar{b}}_{iN}c_{iN}\Gamma \\ \vdots & \vdots & \ddots & \vdots \\ {}* & * & \cdots & \sum_{i=1}^{N}{\bar{b}}_{iN}c_{iN}^{2}\Gamma^{T}P_{i}\Gamma \end{array}\right]$.*

*Then, the observer gains can be determined by $K_{i}=P_{i}^{-1}S_{i}$.*

**Proof** **Theorem** **1.** Choose the following Lyapunov function:

(7) $$V\left(k\right)=\sum_{i=1}^{N}e_{i,k}^{T}P_{i}e_{i,k}.$$

Deriving the difference of $V\left(k\right)$, one obtains:

(8) $$\begin{array}{c} \Delta V\left(k\right)\\ =\sum_{i=1}^{N}\left(e_{i,k+1}^{T}P_{i}e_{i,k+1}-e_{i,k}^{T}P_{i}e_{i,k}\right)\\ =\sum_{i=1}^{N}\{{\left[{\tilde{f}}_{i,k}+\left(A+K_{i}H_{i}\right)e_{i,k}+d\sum_{j=1}^{N}b_{ij,k}c_{ij}\Gamma e_{j,k}\right]}^{T}P_{i}\left[{\tilde{f}}_{i,k}+\left(A+K_{i}H_{i}\right)e_{i,k}+d\sum_{j=1}^{N}b_{ij,k}c_{ij}\Gamma e_{j,k}\right]\\ \;\;\;\;-e_{i,k}^{T}P_{i}e_{i,k}\}\\ =\sum_{i=1}^{N}\{{\tilde{f}}_{i,k}^{T}P_{i}{\tilde{f}}_{i,k}+{\tilde{f}}_{i,k}^{T}P_{i}\left(A+K_{i}H_{i}\right)e_{i,k}+e_{i,k}^{T}{\left(A+K_{i}H_{i}\right)}^{T}P_{i}{\tilde{f}}_{i,k}+{\tilde{f}}_{i,k}^{T}P_{i}d\sum_{j=1}^{N}b_{ij,k}c_{ij}\Gamma e_{j,k}\\ \;\;\;\;+d{\left[\sum_{j=1}^{N}b_{ij,k}c_{ij}\Gamma e_{j,k}\right]}^{T}P_{i}{\tilde{f}}_{i,k}+e_{i,k}^{T}{\left(A+K_{i}H_{i}\right)}^{T}P_{i}\left(A+K_{i}H_{i}\right)e_{i,k}\\ \;\;\;\;+e_{i,k}^{T}{\left(A+K_{i}H_{i}\right)}^{T}P_{i}d\sum_{j=1}^{N}b_{ij,k}c_{ij}\Gamma e_{j,k}+d{\left[\sum_{j=1}^{N}b_{ij,k}c_{ij}\Gamma e_{j,k}\right]}^{T}P_{i}\left(A+K_{i}H_{i}\right)e_{i,k}\\ \;\;\;\;+d^{2}{\left[\sum_{j=1}^{N}b_{ij,k}c_{ij}\Gamma e_{j,k}\right]}^{T}P_{i}\sum_{j=1}^{N}b_{ij,k}c_{ij}\Gamma e_{j,k}-e_{i,k}^{T}P_{i}e_{i,k}\}. \end{array}$$

Let $\Pi =diag\left(H_{1}^{T}S_{1}^{T}P_{1}^{-1}S_{1}H_{1},\ldots ,H_{N}^{T}S_{N}^{T}P_{N}^{-1}S_{N}H_{N}\right)$, $e_{k}={\left[e_{1,k}^{T}\ldots e_{N,k}^{T}\right]}^{T}$, ${\tilde{f}}_{k}={\left[{\tilde{f}}_{1,k}^{T}\ldots {\tilde{f}}_{N,k}^{T}\right]}^{T}$, and $\eta_{k}={\left[e_{k}^{T}\;\;\;{\tilde{f}}_{k}^{T}\right]}^{T}$. Taking the mathematical expectation of $\Delta V\left(k\right)$, one has:

(9) $$\begin{array}{cc} E\left[\Delta V\left(k\right)\right] & ={\tilde{f}}_{k}^{T}\Lambda {\tilde{f}}_{k}+{\tilde{f}}_{k}^{T}M^{T}e_{k}+e_{k}^{T}M{\tilde{f}}_{k}+{\tilde{f}}_{k}^{T}d\Lambda Ge_{k}\\ & \;\;\;\;+e_{k}^{T}dG^{T}\Lambda {\tilde{f}}_{k}+e_{k}^{T}\left(\Pi +{\bar{A}}^{T}\Lambda \bar{A}+Q\bar{A}+{\bar{A}}^{T}Q^{T}\right)e_{k}\\ & \;\;\;\;+e_{k}^{T}\left(dMG+dG^{T}M^{T}\right)e_{k}+e_{k}^{T}d^{2}\Phi e_{k}-e_{k}^{T}\Lambda e_{k}\\ & =\eta_{k}^{T}\left[\begin{array}{cc} \Pi +\Psi & M+dG^{T}\Lambda \\ M^{T}+d\Lambda G & \Lambda \end{array}\right]\eta_{k}. \end{array}$$

Then, from the Lipschitz condition (Assumption 1), we can get that ${\tilde{f}}_{k}^{T}{\tilde{f}}_{k}\leq \alpha^{2}e_{k}^{T}e_{k}$, which is equivalent to

(10) $$T_{k}=\eta_{k}^{T}\left[\begin{array}{cc} -\alpha^{2}I_{N\cdot n} & O\\ O & I_{N\cdot n} \end{array}\right]\eta_{k}\leq 0.$$

As the $T_{k}$ is non-positive, (9) is negative definite if and only if there exists a scalar τ>0 such that $E\left[\Delta V\left(k\right)\right]<\tau T_{k}$. Hence, the following inequality can be obtained:

(11) $$\left[\begin{array}{cc} \Pi +\Psi +\tau \alpha^{2}I_{N\cdot n} & M+dG^{T}\Lambda \\ M^{T}+d\Lambda G & \Lambda -\tau I_{N\cdot n} \end{array}\right]<0.$$

Using Lemma 1, we can see that (11) is equivalent to (6).

According to the Lyapunov stability theory and stochastic analysis, the error system (5) is asymptotically stable (i.e., the error variables will converge to zero), and the observer gains can be obtained by $K_{i}=P_{i}^{-1}S_{i}$. The proof is completed. □

We can calculate the observer gains $K_{i}$ by solving the LMI (6) and complete the construction of the proposed complex dynamical network model (3).

**Remark** **5.** *Many works [5,6,7,34,35,36,37,38] have assumed state-coupling (usually diagonal coupling) among the nodes in a network, implying that a node communicates with its connected neighbors by all its state variables. In addition, there exists the output-coupling pattern that each node communicates with neighbors only by its outputs. Here, we generalize the proposed modeling method to a output-coupling complex dynamical network with random data loss:*

(12) $$\begin{array}{c} x_{i,k+1}\;=\;Ax_{i,k}+f\left(x_{i,k}\right)+d\sum_{j=1}^{N}\left[b_{ij,k}c_{ij}Ly_{j,k}+\left(1-b_{ij,k}\right)c_{ij}L{\hat{y}}_{j,k}\right],\\ y_{i,k}\;=\;H_{i}x_{i,k},\\ {\hat{x}}_{i,k+1}\;=\;A{\hat{x}}_{i,k}+f\left({\hat{x}}_{i,k}\right)+d\sum_{j=1}^{N}c_{ij}L{\hat{y}}_{j,k}+K_{i}\left({\hat{y}}_{i,k}-y_{i,k}\right),\\ {\hat{y}}_{i,k}\;=\;H_{i}{\hat{x}}_{i,k}, \end{array}$$

*where $L\in R^{n\times m}$ denotes the inner coupling matrix.*

*The analysis concept of the output-coupling complex dynamical network (12) is similar to that of the proposed model (3), so the detailed analysis is omitted here.*

## 4. Numerical Simulation

In this section, we give two numerical examples to demonstrate the validity of the proposed discrete-time complex dynamical network model with random data loss. Here, we consider complex dynamical networks generated from the WS small-world network model and the BA scale-free network model due to the universality of the “small-world” and “scale-free” characteristics in most real networks.

**Example** **1.** *A state-coupling WS small-world network.*

**Figure 1:**
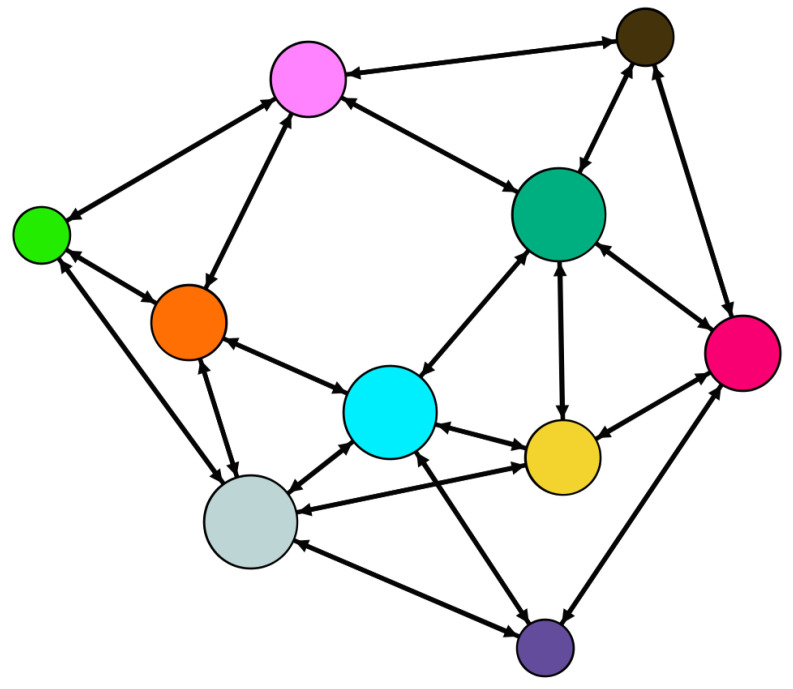
Topology structure of the WS small-world network (the size of node depends on its degree).

Consider the state-coupling WS small-world network with 10 nodes shown in Figure 1. The corresponding network coupling configuration matrix is:

$$C=\left[\begin{array}{cccccccccc} -4 & 1 & 1 & 0 & 0 & 0 & 0 & 0 & 1 & 1\\ 1 & -3 & 1 & 0 & 0 & 0 & 0 & 0 & 1 & 0\\ 1 & 1 & -4 & 1 & 1 & 0 & 0 & 0 & 0 & 0\\ 0 & 0 & 1 & -3 & 1 & 1 & 0 & 0 & 0 & 0\\ 0 & 0 & 1 & 1 & -5 & 1 & 1 & 0 & 0 & 1\\ 0 & 0 & 0 & 1 & 1 & -4 & 1 & 1 & 0 & 0\\ 0 & 0 & 0 & 0 & 1 & 1 & -4 & 0 & 1 & 1\\ 0 & 0 & 0 & 0 & 0 & 1 & 0 & -3 & 1 & 1\\ 1 & 1 & 0 & 0 & 0 & 0 & 1 & 1 & -5 & 1\\ 1 & 0 & 0 & 0 & 1 & 0 & 1 & 1 & 1 & -5 \end{array}\right].$$

The node dynamic is the following nonlinear system:

(13) $$\left\{\begin{array}{c} x_{k+1}=-y_{k}+0.02e^{-x_{k}^{2}},\\ y_{k+1}=x_{k}+0.199y_{k},\\ z_{k+1}=x_{k}-5.7-0.02e^{-z_{k}^{2}}. \end{array}\right.$$

**Figure 2:**
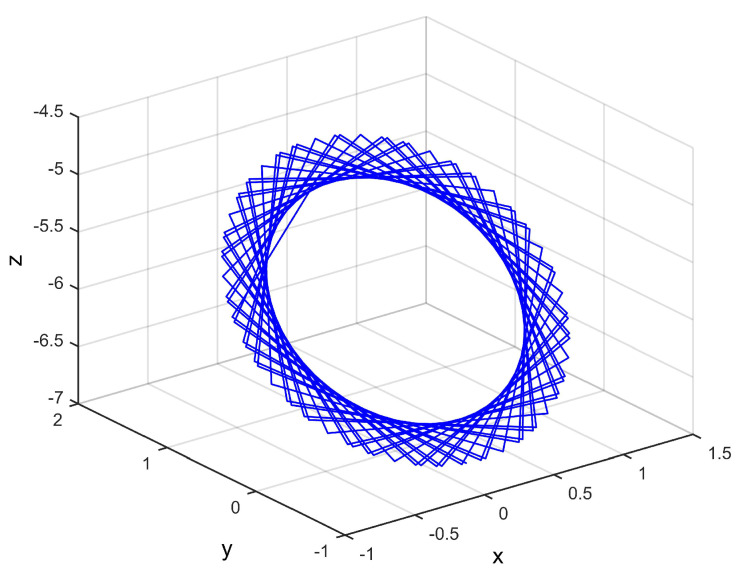
Phase diagram of the isolated node.

This satisfies Assumption 1 by α=0.4. From Figure 2, we know this node dynamic is a non-stationary system.

The simulation parameters are as follows:

d=0.01, τ=1, $A=\left[\begin{array}{ccc} 0 & -1 & 0\\ 1 & 0.199 & 0\\ 1 & 0 & 0 \end{array}\right]$, $\Gamma =\left[\begin{array}{ccc} 1 & 0 & 0\\ 0 & 1 & 0\\ 0 & 0 & 1 \end{array}\right]$,

$H_{1}=\left[\begin{array}{ccc} 0.9 & 0 & 0 \end{array}\right]$, $H_{2}=\left[\begin{array}{ccc} 0.5 & 0 & 0 \end{array}\right]$, $H_{3}=\left[\begin{array}{ccc} 0.8 & 0 & 0 \end{array}\right]$, $H_{4}=\left[\begin{array}{ccc} 0.7 & 0 & 0 \end{array}\right]$, $H_{5}=\left[\begin{array}{ccc} 0.9 & 0 & 0 \end{array}\right]$,

$H_{6}=\left[\begin{array}{ccc} 0.6 & 0 & 0 \end{array}\right]$, $H_{7}=\left[\begin{array}{ccc} 0.7 & 0 & 0 \end{array}\right]$, $H_{8}=\left[\begin{array}{ccc} 0.8 & 0 & 0 \end{array}\right]$, $H_{9}=\left[\begin{array}{ccc} 0.9 & 0 & 0 \end{array}\right]$, $H_{10}=\left[\begin{array}{ccc} 0.5 & 0 & 0 \end{array}\right]$,

$\bar{B}=\left[\begin{array}{cccccccccc} 1 & 0.8 & 0.7 & 0.6 & 0.8 & 0.8 & 0.7 & 0.6 & 0.6 & 0.8\\ 0.6 & 1 & 0.7 & 0.8 & 0.6 & 0.8 & 0.6 & 0.7 & 0.8 & 0.6\\ 0.6 & 0.7 & 1 & 0.6 & 0.6 & 0.7 & 0.8 & 0.8 & 0.7 & 0.8\\ 0.8 & 0.6 & 0.7 & 1 & 0.7 & 0.6 & 0.8 & 0.8 & 0.6 & 0.6\\ 0.6 & 0.7 & 0.8 & 0.6 & 1 & 0.6 & 0.7 & 0.7 & 0.7 & 0.8\\ 0.8 & 0.7 & 0.7 & 0.8 & 0.6 & 1 & 0.7 & 0.7 & 0.7 & 0.6\\ 0.8 & 0.8 & 0.8 & 0.6 & 0.6 & 0.7 & 1 & 0.7 & 0.6 & 0.7\\ 0.7 & 0.6 & 0.8 & 0.7 & 0.6 & 0.7 & 0.7 & 1 & 0.6 & 0.8\\ 0.6 & 0.6 & 0.8 & 0.6 & 0.7 & 0.8 & 0.7 & 0.6 & 1 & 0.7\\ 0.7 & 0.7 & 0.8 & 0.8 & 0.6 & 0.8 & 0.7 & 0.8 & 0.6 & 1 \end{array}\right]$. 

The initial conditions of $x_{i,k},{\hat{x}}_{i,k}\left(i=1,2,\ldots ,10\right)$ are respectively taken as the random numbers in the intervals $\left[-2,\;2\right]$ and $\left[-3,\;3\right]$. Then, according to Theorem 1 and using the YALMIP toolbox in Matlab, we can get the $P_{i}$ and the corresponding observer gains $K_{i}$ as follows:

$P_{1}=\left[\begin{array}{ccc} 0.2811 & 0.0813 & -0.0066\\ 0.0813 & 0.7521 & -0.0302\\ -0.0066 & -0.0302 & 0.6318 \end{array}\right]$, $P_{2}=\left[\begin{array}{ccc} 0.2816 & 0.0821 & -0.0067\\ 0.0821 & 0.7525 & -0.0302\\ -0.0067 & -0.0302 & 0.6320 \end{array}\right]$,

$P_{3}=\left[\begin{array}{ccc} 0.2804 & 0.0795 & -0.0065\\ 0.0795 & 0.7510 & -0.0301\\ -0.0065 & -0.0301 & 0.6309 \end{array}\right]$, $P_{4}=\left[\begin{array}{ccc} 0.2814 & 0.0817 & -0.0066\\ 0.0817 & 0.7523 & -0.0302\\ -0.0066 & -0.0302 & 0.6319 \end{array}\right]$,

$P_{5}=\left[\begin{array}{ccc} 0.2802 & 0.0796 & -0.0065\\ 0.0796 & 0.7510 & -0.0301\\ -0.0065 & -0.0301 & 0.6310 \end{array}\right]$, $P_{6}=\left[\begin{array}{ccc} 0.2810 & 0.0808 & -0.0066\\ 0.0808 & 0.7519 & -0.0302\\ -0.0066 & -0.0302 & 0.6316 \end{array}\right]$,

$P_{7}=\left[\begin{array}{ccc} 0.2806 & 0.0800 & -0.0065\\ 0.0800 & 0.7513 & -0.0302\\ -0.0065 & -0.0302 & 0.6312 \end{array}\right]$, $P_{8}=\left[\begin{array}{ccc} 0.2816 & 0.0821 & -0.0067\\ 0.0821 & 0.7525 & -0.0302\\ -0.0067 & -0.0302 & 0.6320 \end{array}\right]$,

$P_{9}=\left[\begin{array}{ccc} 0.2796 & 0.0783 & -0.0064\\ 0.0783 & 0.7499 & -0.0301\\ -0.0064 & -0.0301 & 0.6302 \end{array}\right]$, $P_{10}=\left[\begin{array}{ccc} 0.2800 & 0.0791 & -0.0064\\ 0.0791 & 0.7507 & -0.0301\\ -0.0064 & -0.0301 & 0.6308 \end{array}\right]$, 

$K_{1}=\left[\begin{array}{c} -0.1208\\ -1.0299\\ -1.0316 \end{array}\right]$, $K_{2}=\left[\begin{array}{c} -0.2235\\ -1.8531\\ -1.8564 \end{array}\right]$, $K_{3}=\left[\begin{array}{c} -0.1280\\ -1.1595\\ -1.1608 \end{array}\right]$, $K_{4}=\left[\begin{array}{c} -0.1574\\ -1.3238\\ -1.3261 \end{array}\right]$, $K_{5}=\left[\begin{array}{c} -0.1139\\ -1.0307\\ -1.0319 \end{array}\right]$,

$K_{6}=\left[\begin{array}{c} -0.1785\\ -1.5451\\ -1.5474 \end{array}\right]$, $K_{7}=\left[\begin{array}{c} -0.1485\\ -1.3249\\ -1.3265 \end{array}\right]$, $K_{8}=\left[\begin{array}{c} -0.1397\\ -1.1582\\ -1.1603 \end{array}\right]$, $K_{9}=\left[\begin{array}{c} -0.1087\\ -1.0313\\ -1.0322 \end{array}\right]$, $K_{10}=\left[\begin{array}{c} -0.2019\\ -1.8557\\ -1.8577 \end{array}\right]$. 

**Figure 3:**
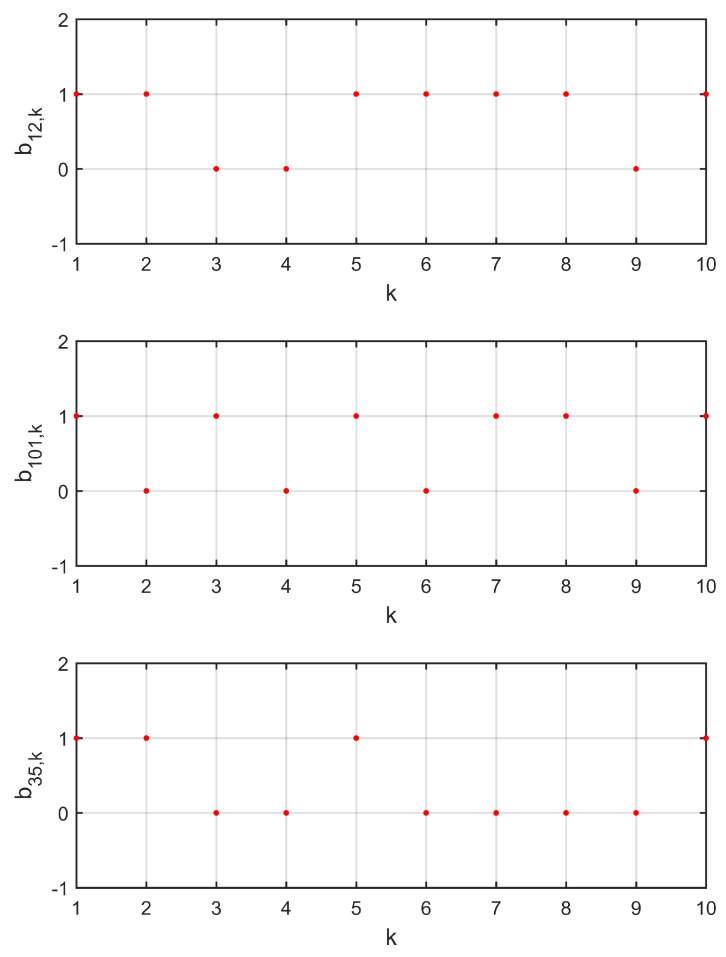
Evolutions of the random process $b_{12,k}$, $b_{101,k}$, and $b_{35,k}$ in Example 1. ${\hat{b}}_{12}=0.2$, ${\hat{b}}_{101}=0.3$, and ${\hat{b}}_{35}=0.4$.

**Figure 4:**
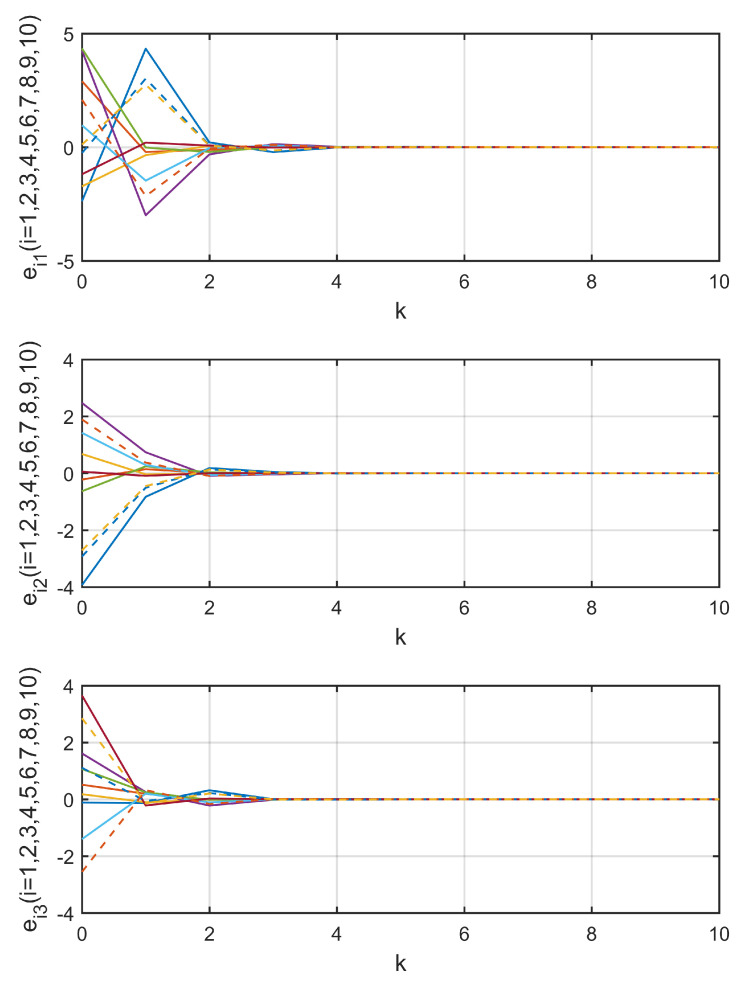
Trajectories of observation errors in Example 1.

Three data transmission channels $c_{12}$, $c_{101}$, and $c_{35}$ were chosen to show the process of random data loss, as shown in Figure 3. Figure 4 shows the trajectories of observation errors $e_{in,k}\left(i=1,2,\ldots ,10;n=1,2,3\right)$ in Example 1. It can be observed that all of the observation errors converged to zero after the step k=4, that is, the observer states ${\hat{x}}_{i,k}$ approached the network states $x_{i,k}$, which implies the lost data was compensated without bias. Along with Figure 3, it can be said that the influence of random data loss was eliminated, even if there were still data losses after k=4.

**Example** **2.** *A state-coupling BA scale-free network.*

**Figure 5:**
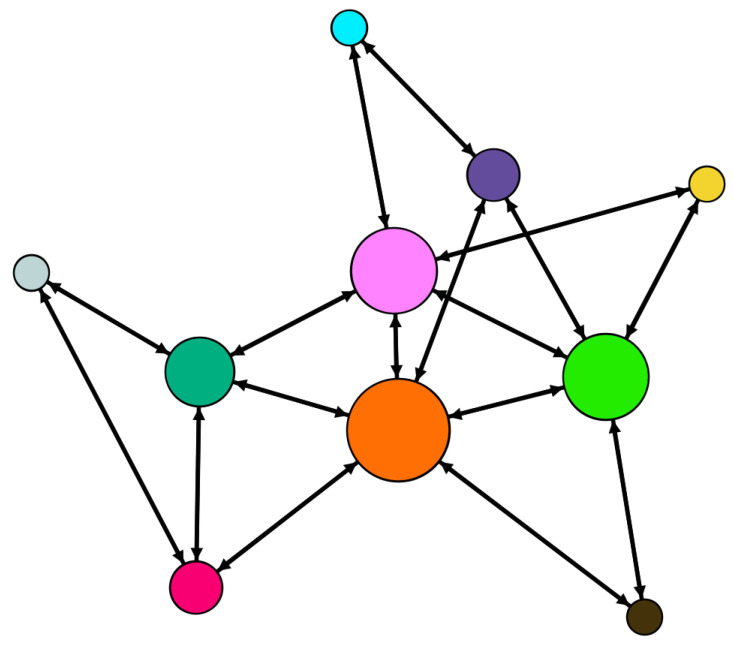
Topology structure of the BA scale-free small-world network (the size of node depends on its degree).

Consider a state-coupling BA scale-free network with 10 nodes shown in Figure 5. The corresponding network coupling configuration matrix is

$$C=\left[\begin{array}{cccccccccc} -6 & 1 & 1 & 1 & 1 & 1 & 0 & 1 & 0 & 0\\ 1 & -5 & 1 & 1 & 0 & 0 & 1 & 1 & 0 & 0\\ 1 & 1 & -5 & 0 & 1 & 0 & 1 & 0 & 0 & 1\\ 1 & 1 & 0 & -2 & 0 & 0 & 0 & 0 & 0 & 0\\ 1 & 0 & 1 & 0 & -4 & 1 & 0 & 0 & 1 & 0\\ 1 & 0 & 0 & 0 & 1 & -3 & 0 & 0 & 1 & 0\\ 0 & 1 & 1 & 0 & 0 & 0 & -2 & 0 & 0 & 0\\ 1 & 1 & 0 & 0 & 0 & 0 & 0 & -3 & 0 & 1\\ 0 & 0 & 0 & 0 & 1 & 1 & 0 & 0 & -2 & 0\\ 0 & 0 & 1 & 0 & 0 & 0 & 0 & 1 & 0 & -2 \end{array}\right].$$

The output matrices are as follows:

$H_{1}=\left[\begin{array}{ccc} 0.7 & 0.5 & 0.4 \end{array}\right]$, $H_{2}=\left[\begin{array}{ccc} 0.5 & 0.6 & 0.8 \end{array}\right]$, $H_{3}=\left[\begin{array}{ccc} 0.6 & 0.3 & 0.7 \end{array}\right]$, $H_{4}=\left[\begin{array}{ccc} 0.8 & 0.7 & 0.8 \end{array}\right]$,

$H_{5}=\left[\begin{array}{ccc} 0.7 & 0.6 & 0.9 \end{array}\right]$, $H_{6}=\left[\begin{array}{ccc} 0.8 & 0.5 & 0.6 \end{array}\right]$, $H_{7}=\left[\begin{array}{ccc} 0.7 & 0.3 & 0.4 \end{array}\right]$, $H_{8}=\left[\begin{array}{ccc} 0.4 & 0.7 & 0.6 \end{array}\right]$,

$H_{9}=\left[\begin{array}{ccc} 0.7 & 0.8 & 0.5 \end{array}\right]$, $H_{10}=\left[\begin{array}{ccc} 0.9 & 0.7 & 0.7 \end{array}\right]$. 

The node dynamic and other parameters are consistent with Example 1, and the initial conditions of $x_{i,k},{\hat{x}}_{i,k}$ are respectively taken as the random numbers in the intervals $\left[-2,\;2\right]$ and $\left[-5,\;5\right]$. Then, $P_{i}$ and the corresponding observer gains $K_{i}$ are obtained as follows:

$P_{1}=\left[\begin{array}{ccc} 0.4464 & -0.0838 & -0.1230\\ -0.0838 & 0.6130 & -0.2751\\ -0.1230 & -0.2751 & 0.5101 \end{array}\right]$, $P_{2}=\left[\begin{array}{ccc} 0.4969 & -0.0283 & -0.0976\\ -0.0283 & 0.5941 & -0.3758\\ -0.0976 & -0.3758 & 0.5415 \end{array}\right]$,

$P_{3}=\left[\begin{array}{ccc} 0.4003 & -0.0292 & -0.1258\\ -0.0292 & 0.6153 & -0.3620\\ -0.1258 & -0.3620 & 0.5533 \end{array}\right]$, $P_{4}=\left[\begin{array}{ccc} 0.4801 & -0.0515 & -0.1353\\ -0.0515 & 0.6071 & -0.3338\\ -0.1353 & -0.3338 & 0.5503 \end{array}\right]$,

$P_{5}=\left[\begin{array}{ccc} 0.4632 & -0.0350 & -0.1150\\ -0.0350 & 0.6042 & -0.3641\\ -0.1150 & -0.3641 & 0.5441 \end{array}\right]$, $P_{6}=\left[\begin{array}{ccc} 0.4313 & -0.0604 & -0.1420\\ -0.0604 & 0.6237 & -0.3018\\ -0.1420 & -0.3018 & 0.5479 \end{array}\right]$,

$P_{7}=\left[\begin{array}{ccc} 0.3794 & -0.0580 & -0.1588\\ -0.0580 & 0.6603 & -0.2462\\ -0.1588 & -0.2462 & 0.5998 \end{array}\right]$, $P_{8}=\left[\begin{array}{ccc} 0.5676 & -0.0362 & -0.0920\\ -0.0362 & 0.5813 & -0.3573\\ -0.0920 & -0.3573 & 0.5331 \end{array}\right]$,

$P_{9}=\left[\begin{array}{ccc} 0.5581 & -0.0876 & -0.1422\\ -0.0876 & 0.6048 & -0.2932\\ -0.1422 & -0.2932 & 0.5456 \end{array}\right]$, $P_{10}=\left[\begin{array}{ccc} 0.4707 & -0.0677 & -0.1525\\ -0.0677 & 0.6188 & -0.3005\\ -0.1525 & -0.3005 & 0.5629 \end{array}\right]$, 

$K_{1}=\left[\begin{array}{c} 0.5998\\ -0.6775\\ -0.5570 \end{array}\right]$, $K_{2}=\left[\begin{array}{c} 0.5882\\ -0.3431\\ -0.2201 \end{array}\right]$, $K_{3}=\left[\begin{array}{c} 0.6326\\ -0.5889\\ -0.4611 \end{array}\right]$, $K_{4}=\left[\begin{array}{c} 0.4555\\ -0.4020\\ -0.3082 \end{array}\right]$, $K_{5}=\left[\begin{array}{c} 0.4927\\ -0.3635\\ -0.2615 \end{array}\right]$,

$K_{6}=\left[\begin{array}{c} 0.4888\\ -0.5764\\ -0.4776 \end{array}\right]$, $K_{7}=\left[\begin{array}{c} 0.4757\\ -0.9462\\ -0.8544 \end{array}\right]$, $K_{8}=\left[\begin{array}{c} 0.6635\\ -0.3466\\ -0.2053 \end{array}\right]$, $K_{9}=\left[\begin{array}{c} 0.5371\\ -0.4788\\ -0.3681 \end{array}\right]$, $K_{10}=\left[\begin{array}{c} 0.4237\\ -0.4685\\ -0.3825 \end{array}\right]$. 

**Figure 6:**
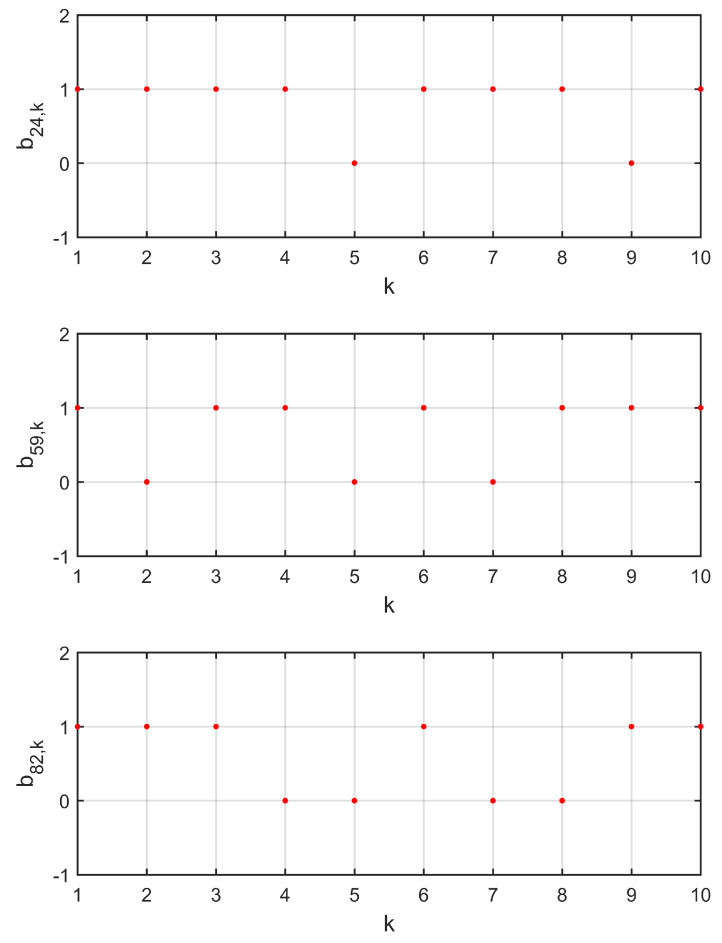
Evolutions of the random process $b_{24,k}$, $b_{59,k}$, and $b_{82,k}$ in Example 2. ${\hat{b}}_{24}=0.2$, ${\hat{b}}_{59}=0.3$, and ${\hat{b}}_{82}=0.4$.

**Figure 7:**
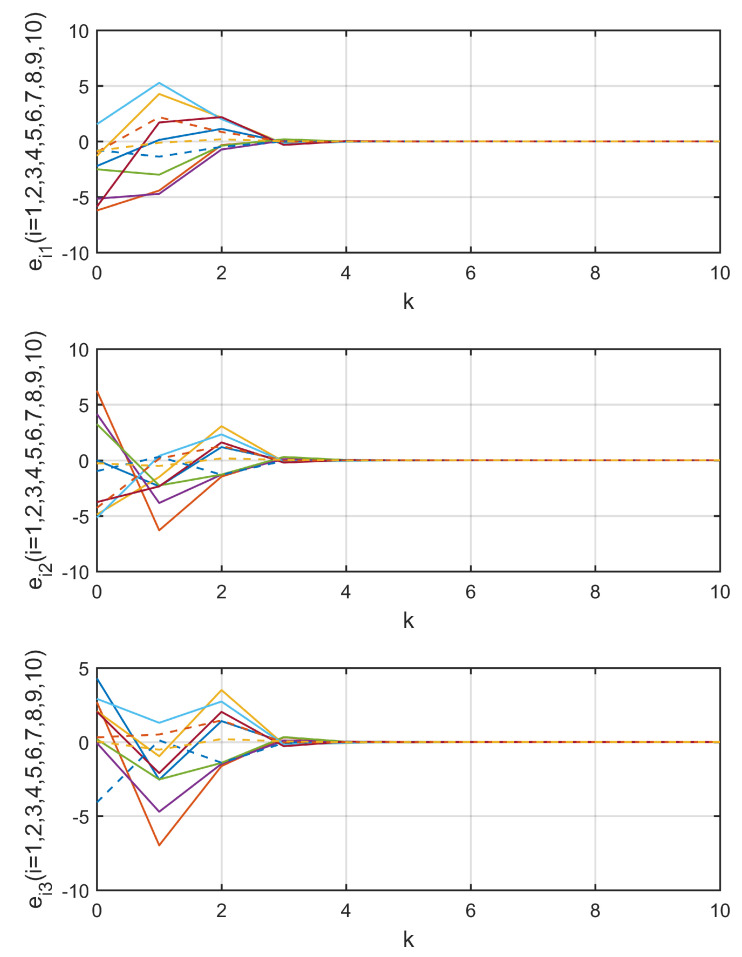
Trajectories of observation errors in Example 2.

Three data transmission channels $c_{24}$, $c_{59}$, and $c_{82}$ were chosen to show the process of random data loss in Figure 6. Along with the trajectories of observation errors $e_{in,k}\left(i=1,2,\ldots ,10;n=1,2,3\right)$ in Example 2, which are shown in Figure 7, it can be observed that the observation errors all converged to zero after step k=4, that is, the observer states ${\hat{x}}_{i,k}$ approached the network states $x_{i,k}$, which implies the lost data was compensated without bias and the influence of random data loss was eliminated, even if there were still data losses after k=4.

The simulation examples above cover the networks of the WS small-world and the BA scale-free topology structures, indicating that the proposed modeling method is effective in eliminating the influence of random data loss in complex dynamical networks.

## 5. Conclusions

In this paper, we proposed a new model for discrete-time complex dynamical networks with random data losses which may occur in the links between every two neighbor nodes. The data losses are described as a set of random variables satisfying a Bernoulli distribution. To construct the proposed model, an auxiliary observer was introduced, and we chose the observer states to compensate for the lost states in the coupling term. According to the analysis, we derived a sufficient condition to guarantee the compensation values finally equal to the lost values, thus the proposed model finally succeeded in eliminating the influence of data loss. From the simulation results, the proposed model was demonstrated to be effective.

In this paper, we consider only the internal data loss happening on the interaction topology in complex dynamical networks. However, it is possible to have data loss in the transmission of output variables from the observed network to the controller in the observer simultaneously. Further study could focus on ways to solve this problem. It is also interesting to mention that a number of systems and networks possess multiple time scales [39,40,41,42], where the data loss could happen on different time scales. To extend our model to complex dynamical networks where different node systems have different time scales is thus an interesting research topic. In addition, we only focused on the asymptotic convergence of the error system, and we will study the finite-time and the fixed-time asymptotic convergence in future work.
